# Displaced femoral neck fracture in a pregnant patient diagnosed with transient osteoporosis of the hip

**DOI:** 10.1051/sicotj/2022045

**Published:** 2022-11-25

**Authors:** Shai Factor, Juan Barriga, Dania Halperin, Raphael Krespi, Tomer Ben-Tov

**Affiliations:** Department of Orthopaedic Surgery, Tel Aviv Medical Center, Affiliated with the Sackler Faculty of Medicine, Tel Aviv University Tel Aviv Israel

**Keywords:** Transient osteoporosis, Idiopathic, Pregnancy, Femoral neck fracture, Surgery

## Abstract

Transient osteoporosis of pregnancy (TOP) is a self-limiting pathology with unspecified etiology. It is typically found in women in late pregnancy or early postpartum. A femoral neck fracture is an infrequent complication. Herein, we describe a TOP case in a 38-year-old female who suffered a displaced sub-capital femoral neck fracture without obvious trauma at 28 weeks of gestation. The patient underwent operative treatment using closed reduction and internal fixation (CRIF), using cannulated screws, with no intraoperative complications. The postoperative radiograph revealed a collapse and further displacement of the femoral neck. A decision was made to postpone a definitive treatment to a postpartum date. The patient underwent a cesarean section at 38-week of gestation with no complications. At her latest follow-up, 24 months postoperatively, the patient was asymptomatic. Pelvic and hip radiographs demonstrated consolidation of the fracture. Level of evidence: IV.

## Introduction

Musculoskeletal (MSK) signs such as hip, pelvis and groin pain are frequent complaints of pregnancy. Joint laxity due to hormonal changes, the position of the uterus, and increased weight (neural compression by fluid retention) are the main causes of these complaints [1]. These symptoms are generally treated nonoperatively, without specific diagnosis although there may be cases with severe and progressive pain, particularly during the second and third trimesters of pregnancy [1, 2]. In these cases, transient osteoporosis of pregnancy (TOP) needs to be considered for differential diagnosis [3].

Transient Regional Osteoporosis (TRO) is a self-limited and reversible disorder characterized by rapidly developing localized osteoporosis with no determinate etiology such as trauma or prolonged immobilization [4]. TRO mainly affects the periarticular regions and presents in addition to the severe onset of groin pain and increasing hip pain throughout the third trimester of pregnancy [3]. TOP belongs to the TRO and is a progressively rare condition, primarily unilateral, resulting from impaired venous return with edema in the bone marrow [5]. This defect increases the intramedullary pressure in the affected bone area and is found in middle-aged males and females in late pregnancy or early postpartum. The condition resolves after 6–8 months [3].

Regarding pregnancy-related osteoporosis, femoral neck fracture is uncommon, hence no specific guidelines for treatment have been established and no treatment option is superior [6]. Conversely, when related to trauma, it is recommended that extremity-closed fractures be treated conservatively when suitable, and the surgery should be performed after the labor until postpartum [2, 7].

## Case report

**Figure 1:**
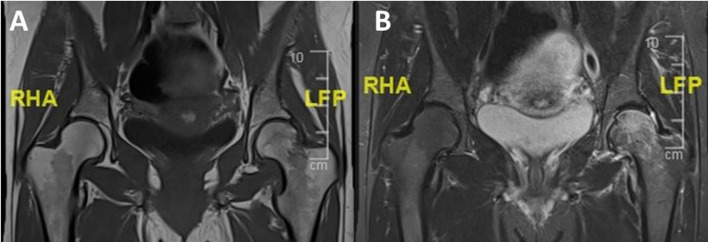
MRI performed at 27 weeks of gestation showed a decreased signal intensity in the proximal femur on T1-weighted imaging (A) and an increased signal on the T2-weighted imaging sequence. (B) MRI; Magnetic Resonance Imaging.

A 38-year-old gravida 3, para 0 woman, presented to the emergency department due to sudden and severe pain in her left groin, without a history of current trauma. The patient was currently at 28 weeks of her third pregnancy, with no pathologies and normal results in the amniocentesis after the first 18 weeks. History revealed ten pack-years of chronic smoking, and two spontaneous miscarriages: the first not well documented and the second one in the 7th week of pregnancy. Previous medical workup did not reveal any coagulopathy. During her 26 weeks of gestation, the patient suffered from hip pain, first, she was treated with oral analgesics with no improvement and limitations in her daily activity. She underwent Magnetic Resonance Imaging (MRI) and was diagnosed with left TOP at week 27 with no fracture (Fig. 1). The patient was instructed to bear weight as tolerated, avoid strenuous physical activity, and continue orthopedic follow-up in an ambulatory setting.

**Figure 2:**
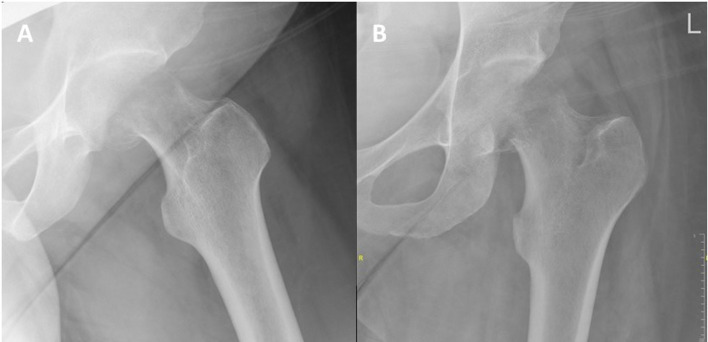
Lateral (A) and anteroposterior (B) radiographs of the left hip show a displaced sub-capital femoral neck fracture.

Her physical examination consisted of an externally rotated and shortened left leg. Positive leg roll and axial load and tenderness in the inguinal region during passive motion. She was unable to bear weight on her affected leg and was neurovascular intact. The patient underwent a pelvic and hip radiograph which demonstrated a displaced sub-capital femur fracture of the left hip (Fig. 2).

**Figure 3:**
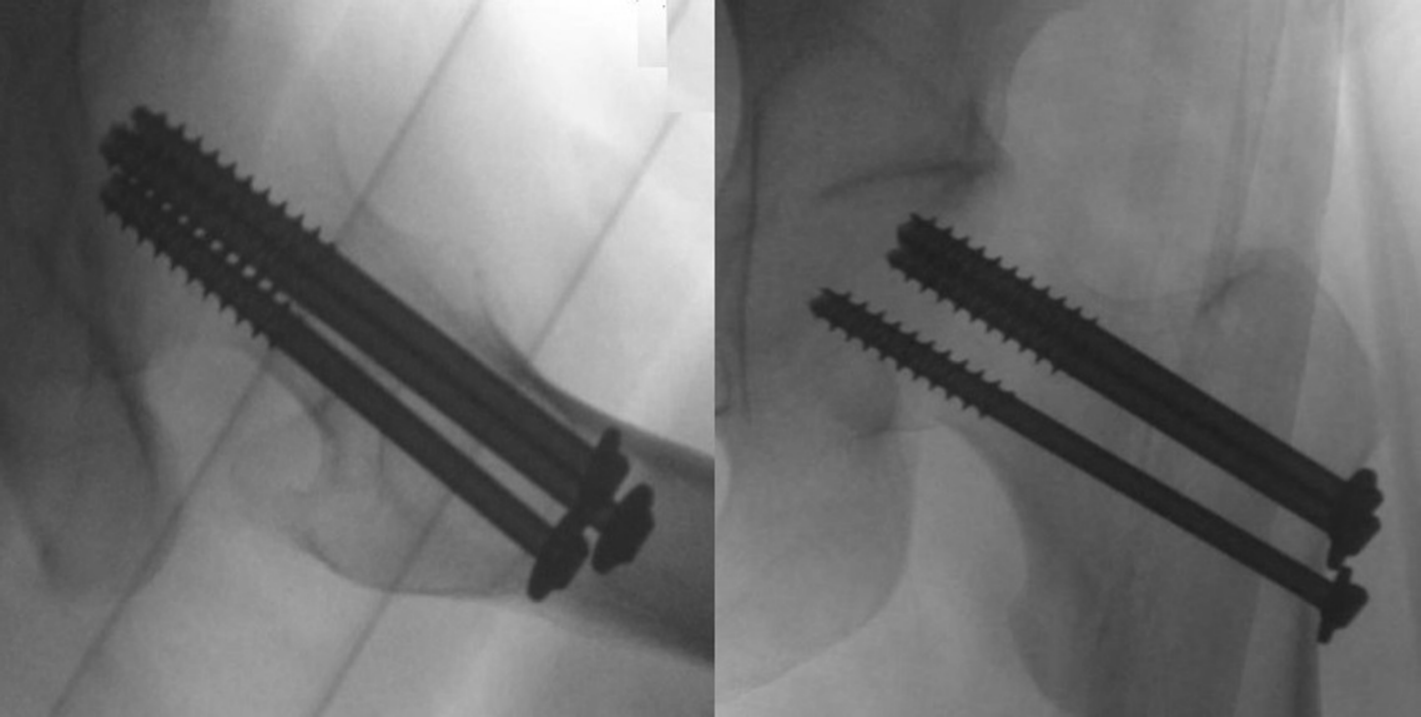
Intraoperative fluoroscopy demonstrate adequate reduction and position of the cannulated screws.

The patient was taken to the operating bay the next day; under general anesthesia, she underwent closed reduction on a traction table. The reduction was performed according to the Leadbetter maneuver [8] and consisted of hip flexion to 45°, followed by gentle internal rotation and sustained traction, in line with the femur. After reduction was confirmed with fluoroscopy, internal fixation of the left femur with three 7.3 mm partially threaded (32 mm), cannulated screws (©DePuy Synthes, Johnson & Johnson, USA) was done. The lengths of the screws were 80 mm & 85 mm for the upper ones and 90 mm for the inferiors one (Fig. 3). The senior author performed the procedure in sixty-four minutes, with no intraoperative complications. After the surgery, she was advised to start her rehabilitation with partial weight-bearing and underwent a single physiotherapy practice during hospitalization. The postoperative radiographic imaging was done one day after the surgery and revealed a collapsed femoral neck, with further displacement. A multidisciplinary discussion was done, including orthopedic and joint replacement reconstruction units, the gynecologist, and the patient herself.

The treatment options were: (1) continue observation with non-weight bearing on her operated leg, consequent radiographs, and surgery if needed after birth; (2) revision of the fixation with the use of a bone graft and (3) total hip replacement. The patient decided to continue with observation and consider surgery postpartum, with a non-weight-bearing protocol until then. She underwent a cesarean by week 38, with no complications. An X-ray was done as part of the consultation one day after the surgery, showing a consolidation of the fracture.

**Figure 4:**
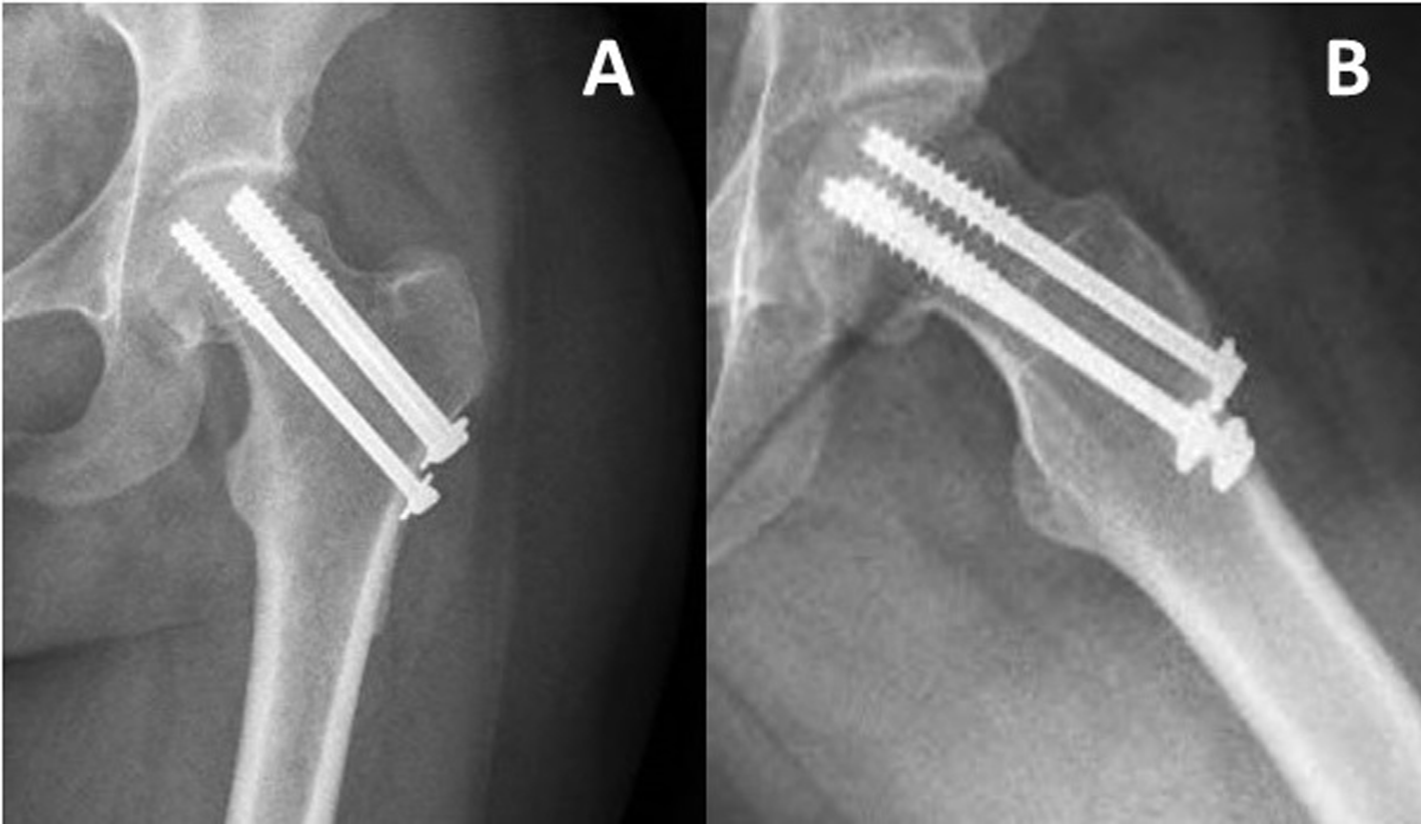
Anteroposterior (A) and lateral (B) radiographs performed on the latest follow-up, 24 months postoperatively, demonstrates consolidation of the fracture with no hardware migration nor intra-articular penetration.

In her most recent follow-up, 24 months following the injury the patient bears weight, with no use of assistive devices (Fig. 4). Due to the proximity of the screws to the articular cartilage and in order to assess the bone condition ideally on MRI, it was recommended to remove the hardware.

## Discussion

Osteonecrosis and TRO of the hip may cause severe hip pain and disability [2]. The differential diagnosis of TOP includes avascular osteonecrosis, infectious or inflammatory processes [9]. Regardless of its benign course, TOP can have major complications during pregnancy, such as stress or insufficiency fractures [3, 10].

In our presented case, the patient had a displaced sub-capital femoral neck fracture at 28 weeks of gestation, she underwent CRIF, using cannulated screws. Postoperative radiograph revealed a collapse and further displacement of the femoral neck. A decision was made to postpone a definitive treatment to a postpartum date. At her latest follow-up, 24 months postoperatively, the patient was asymptomatic. Pelvic and hip radiographs demonstrated consolidation of the fracture.

TOP is a progressively uncommon condition, primarily unilateral, resulting from impaired venous return with edema in the bone marrow [10]. It is usually found in middle-aged males and females in late pregnancy or early postpartum. Mineral deficiencies and defects in fibrinolysis may be risk factors, however, the exact etiology of the condition is not clearly explained [3]. Treatment options of TOP include analgesics and protected weightbearing to prevent pathological fractures [3]. Conservative treatment is appropriate until the symptoms spontaneously resolve, usually up to 8 weeks postpartum [10]. A subsequent femoral neck fracture is not common [9]. Calcitonin and bisphosphonates occasionally shorten recovery time, however, both are category C drugs [11], and the risk versus benefit must be considered. Glucocorticoids, vitamin D, calcium, and NSAIDs, have shown no efficacy [12].

Radiographs can reveal severe osteopenia in advanced cases of TOP [3]. During pregnancy, the preferred imaging modality is Magnetic Resonance Imaging (MRI), which is safer, more sensitive, and effective for identifying skeletal abnormalities, including TOP and hip fractures [7, 13]. Severe hip pain in thinner pregnant women during late pregnancy encourages physicians to perform an MRI. Diffuse bone marrow edema, elevated markers for inflammation, severe and persistent pain in the groin and hip, through late pregnancy are positive findings for TOP [14]. Non or protected-weight bearing is necessary to avoid complications in TOP [15]. Atraumatic fractures may also be observed. Hip fracture is reported during labor; thus, cesarean section is favored in cases with TOP [16, 17].

There is a paucity of data in the literature regarding fractures following TOP (Table 1). In cases where closed extremity fracture occurs due to TOP, the management can be conservative (i.e., nonoperative) or surgical treatment that can be delayed until postpartum when appropriate [18]. Sorbi et al. described [19] a case of a 40-year-old woman, at 36 weeks of pregnancy suffering a displaced midshaft tibia fracture. The patient was first treated with a short leg cast, and underwent surgical treatment, via open reduction and internal fixation (ORIF), postpartum. This case showed that nonoperative modalities can be used effectively when indicated until the postpartum period.

**Table 1:** Sub-capital femoral neck fractures following transient osteoporosis of pregnancy; summary of published cases.^a^

Author, year	Week of gestation	Unilateral\bilateral	Treatment	Outcome
Factor, 2022	28	Unilateral	CRIF with cannulated screws	At 2-year follow-up, the patient was asymptomatic. Pelvic and hip radiographs demonstrated consolidation of the fracture.
Kasahara, 2017	35 + 5	Bilateral	ORIF with cannulated screws	At five months postoperatively, the patient was able to walk without pain.
Emami, 2012	Post-partum	Bilateral	Hemiarthroplasty	At 2-year follow-up, patient was well with satisfactory function.
Vergara-Ferrer, 2011	36	Unilateral	CRIF with cannulated screws	At 11 months postoperatively, the patient was asymptomatic with no impairments of gait, besides a slight limitation of internal rotation.
Guryel, 2010	38	Unilateral	CRIF with cannulated screws	Osteonecrosis of the femoral head.
Cohen, 2007	29	Unilateral	ORIF with cannulated screws	Followed for 24 months with no evidence of osteonecrosis.
Wood, 2003	Post-partum	Unilateral	ORIF with pediculated bone graft and cannulated screws	At 17 months postoperatively, the patient had no groin pain, with a mild limp. Radiographs showed no evidence of osteonecrosis. Subchondral penetration of a superior screw necessitated screw removal.

a Cases published in the last 20 years. ORIF; open reduction internal fixation, CRIF; closed reduction internal fixation.

Fokter et al. [20] presented a case of a patient, that underwent operative treatment 2 weeks after delivery (three weeks following injury). The bone was found to be significantly osteopenic during fixation, therefore, a hip spica was placed postoperatively. The fracture healed uneventfully, showing that osteopenic bone in TOP has normal healing potential. Vergara-Ferrer et al. [10] reviewed 16 cases of hip fracture which all highlighted different therapeutic options (i.e., CRIF or ORIF with cannulated screws; with or without the use of bone graft and partial or total hip arthroplasty). They concluded that there is a wide range of surgical treatment options, in the current literature. Except for one case, in all cases, where an osteosynthesis was performed, the fracture healed with no subsequent necrosis, regardless of the time elapsed since the fracture was detected.

Brodell et al. [21] reported on 2 patients who had a fracture following TOP. One had a healing nondisplaced fracture of the pubic ramus, and the other had a displaced sub-capital fracture of the hip, treated non-operatively. Radiographs of the second patient, performed at 6 weeks following injury, revealed enhanced mineralization of the proximal part of the femur. The patients underwent valgus de-rotation subtrochanteric osteotomy. Both the fracture and the osteotomy healed.

## Conclusion

TOP should be considered for the differential diagnosis of severe hip pain throughout pregnancy, and to prevent further complications, early diagnosis and treatment are essential. In the case of a femoral neck fracture, pregnancy should pose no obstacle to surgical treatment.
